# Cardiovascular Autonomic Function Changes and Predictors During a 2-Year Physical Activity Program in Rheumatoid Arthritis: A PARA 2010 Substudy

**DOI:** 10.3389/fmed.2021.788243

**Published:** 2021-12-15

**Authors:** David Hupin, Philip Sarajlic, Ashwin Venkateshvaran, Cecilia Fridén, Birgitta Nordgren, Christina H. Opava, Ingrid E. Lundberg, Magnus Bäck

**Affiliations:** ^1^Translational Cardiology, Center for Molecular Medicine, Karolinska Institutet, Stockholm, Sweden; ^2^Department of Medicine Solna, Karolinska Institutet, Stockholm, Sweden; ^3^INSERM, U1059, SAINBIOSE, Université de Lyon, Université Jean-Monnet, Saint-Etienne, France; ^4^Department of Clinical and Exercise Physiology, University Hospital of Saint-Etienne, Saint-Etienne, France; ^5^Department of Cardiology, Karolinska University Hospital, Stockholm, Sweden; ^6^Department of Neurobiology, Care Sciences and Society, Karolinska Institutet, Stockholm, Sweden; ^7^Women's Health and Allied Health Professionals Theme, Medical Unit Occupational Therapy and Physiotherapy, Karolinska University Hospital, Stockholm, Sweden; ^8^Rheumatology, Inflammation and Ageing Theme, Karolinska University Hospital, Stockholm, Sweden; ^9^Department of Medicine Solna, Division of Rheumatology, Karolinska Institutet, Stockholm, Sweden

**Keywords:** heart rate recovery, autonomic nervous system, physical activity, rheumatoid arthritis, inflammation, cardiovascular disease, blood pressure, muscular strength

## Abstract

**Background:** Chronic inflammation leads to autonomic dysfunction, which may contribute to the increased risk of cardiovascular diseases (CVD) in patients with rheumatoid arthritis (RA). Exercise is known to restore autonomic nervous system (ANS) activity and particularly its parasympathetic component. A practical clinical tool to assess autonomic function, and in particular parasympathetic tone, is heart rate recovery (HRR). The aim of this substudy from the prospective PARA 2010 study was to determine changes in HRR post-maximal exercise electrocardiogram (ECG) after a 2-year physical activity program and to determine the main predictive factors associated with effects on HRR in RA.

**Methods:** Twenty-five participants performed physiotherapist-guided aerobic and muscle-strengthening exercises for 1 year and were instructed to continue the unsupervised physical activity program autonomously in the next year. All participants were examined at baseline and at years 1 and 2 with a maximal exercise ECG on a cycle ergometer. HRR was measured at 1, 2, 3, 4, and 5 min following peak heart rate during exercise. Machine-learning algorithms with the elastic net linear regression models were performed to predict changes in HRR1 and HRR2 at 1 year and 2 years of the PARA program.

**Results:** Mean age was 60 years, range of 41–73 years (88% women). Both HRR1 and HRR2 increased significantly from baseline to year 1 with guided physical activity and decreased significantly from year 1 to year 2 with unsupervised physical activity. Blood pressure response to exercise, low BMI, and muscular strength were the best predictors of HRR1/HRR2 increase during the first year and HRR1/HRR2 decrease during the second year of the PARA program.

**Conclusion:** ANS activity in RA assessed by HRR was improved by guided physical activity, and machine learning allowed to identify predictors of the HRR response at the different time points. HRR could be a relevant marker of the effectiveness of physical activity recommended in patients with RA at high risk of CVD. Very inactive and/or high CVD risk RA patients may get substantial benefits from a physical activity program.

## Background

The autonomic nervous system (ANS) plays a major role in cardiac and vascular adaptations to environmental stress and is thus a major health marker for the effects of aging and cardiovascular diseases (CVD) (1). Autonomic dysfunction, i.e., increased activity of sympathetic tone and/or less parasympathetic tone at rest is associated with chronic inflammation (2). Rheumatoid arthritis (RA) is the most common inflammatory joint disease affecting 1–2% of the population worldwide (3). Patients with RA exhibit signs of autonomic dysfunction (4, 5), which may contribute to the established vascular (6, 7) and myocardial (3, 8, 9) affection and increased risk of CVD in patients with RA (10–12).

Even if little is known yet about the mechanisms that associate (i) autonomic dysfunction, (ii) chronic inflammation, and (iii) CVD risk in patients with RA, regular physical activity counterbalances these three entities (3, 13). Physical activity is known to restore ANS activity, and particularly its parasympathetic component (14). Also, the vagus nerve, which represents parasympathetic activity, plays a key role in inflammation by regulating the production of cytokines and decreasing circulating inflammatory markers (13). Finally, the increase of parasympathetic activity has been shown to be a strong protective factor against cardiovascular events (1).

A practical clinical tool to assess autonomic function, and in particular parasympathetic tone, is heart rate recovery (HRR) (13, 15, 16). The decrease in heart rate (HR) immediately following a maximal exercise electrocardiogram (ECG) represent reactivation of the parasympathetic tone (17). Vagal reactivation plays an integral part in reducing HR after exercise, especially during the first 2 min (18). A reduced HRR (an indicator of autonomic dysfunction) has been associated with increased risks of both cardiac and all-cause mortality, whereas rapid HR recovery immediately after exercise is associated with a lower risk of CVD and CVD events (17, 19).

Even though autonomic activity has been explored in RA (20) and after an exercise ECG (21, 22), to our knowledge, no study has assessed HRR post-maximal exercise ECG following physical activity intervention in RA. Therefore, the aim of this work was to establish the effects of a 2-year physical activity program (23) on autonomic function in RA, assessed by HRR post-maximal exercise ECG, and to determine the main predictive factors associated with HRR in RA using machine learning algorithms.

## Methods

### Study Population

This is a substudy from the prospective PARA 2010 study (Trial registration number: ISRCTN255 39102) (23). Inclusion criteria were RA according to the American College of Rheumatology, without a history of CVD. For this substudy, 25 PARA 2010 participants were included. These inactive participants, since they did not meet the WHO guidelines on physical activity (24), benefited from a 2-year real-life intervention program. The study protocol was approved by the Stockholm regional ethics committee (reference number 2012/769-32).

### Disease Activity

Disease activity score (DAS28) was measured at baseline from the erythrocyte sedimentation rate (ESR) level, the number of swollen (0–28) and tender joints (0–28), and self-reported general health perception (visual analog scale, VAS 0–10 mm). The DAS28 was scored 0–10 with scores below 3.2 indicating low disease activity and those above 5.1 as high (23). RA activity was also assessed from treatment: disease-modifying antirheumatic drugs (DMARDs), non-steroidal anti-inflammatory drugs, and cortisone. They were recommended to maintain their course of medical treatment during the study.

### Physical Activity Support Program

During the first year, each participant was encouraged to take part in at least two weekly 45 min circuit training sessions (aerobic exercise and muscle strength) with a physiotherapy coach and to perform moderate-to-vigorous physical activity (MVPA) at least 30 min on most days of the week (MVPA ≥ 240 min/week, i.e., ≥12 MET-h/week) using a pedometer for monitoring their number of steps. Physiotherapy coaches initially instructed them on the desired performance of exercises and MVPA and then guided them into support group meetings 1 h every other week to facilitate learning of specific behavioral skills to enable incorporation of physical activity sessions in real life, according to social cognitive theory. Alternative types of physical activity, individually or together with group peers, were encouraged. To prevent relapse during holidays, challenge competitions were organized where participants reported their physical activity and could win simple prizes. Furthermore, they were provided with short message service (SMS) and weekly text messages to monitor and encourage their adherence to the program (23). During the second year, participants were expected to continue the program autonomously. They were encouraged to perform MVPA at least 30 min on most days of the week (MVPA ≥150 min/week, i.e., ≥7.5 MET-h/week) and to report adherence by SMS (23).

### Resting ECG

A standard resting 12-lead ECG was performed after 10 min of rest. From this 10 s electrocardiographic recording, the duration of all normal sinus intervals (RR intervals) was measured using a digital compass application (Compass EP, EP studios, USA). ECGs with <10 normal intervals in a row were excluded. Two intervals following abnormal beats and intervals differing more than 20% from the preceding interval were also considered abnormal and were excluded (25).

For the assessment of ultrashort heart rate variability parameters as surrogates of longer recordings, the square root of the mean squared differences of successive RR intervals (RMSSD in ms) was considered reliable down to 10 s ECG recordings (26, 27).


$$\begin{array}{l} \text{RMSSD}=\sqrt{\frac{1}{N}\overset{N-1}{\underset{i=1}{\sum }}{\left(RR_{i+1}-RR_{i}\right)}^{2}} \end{array}$$

### Exercise ECG

A maximal exercise ECG on the cycle ergometer (Ergomedic, Monark, Sweden) was performed by an experienced operator to rule out cardiac ischemia before starting the program and to assess physical capacity at each visit (baseline, year 1, and year 2). A starting load of 50 W was used with a 20 W/min increase in intensity until exhaustion or any of the relative or absolute contraindications of ACSM's guidelines (28) were met. The results were expressed in “W” and in percent of the predicted value. Continuous ECG was recorded, and blood pressure monitoring was measured throughout the exercise and recovery periods.

Exercise ECG was considered as a maximal cardiac level if participants achieved 90% of their maximal predicted HR (220-age for men and 210-age for women). HR exercise deltas between HR at 2 min from exercise and at rest and between maximal and resting were measured (bpm). The criteria for a positive exercise ECG were previously defined (29, 30).

### Systolic Blood Pressure

Resting blood pressure was measured manually by an experienced nurse using a random-zero sphygmomanometer when the participant was sitting on the cycle ergometer immediately before the exercise phase. Systolic blood pressure (SBP) was measured every 2 min during exercise, and at 2 min and 5 min recovery from exercise. Maximal SBP was the highest value achieved during the exercise ECG. SBP exercise deltas between SBP at 2 min from exercise and at rest and between maximal and resting were measured (mmHg). SBP recovery deltas between maximal and at 2- and 5-min recovery from exercise were also measured (mmHg).

Heart rate recovery was measured at 1, 2, 3, 4, and 5 min following peak HR during exercise. Peak HR was identified as the maximum HR during the exercise protocol. HRR 1 min (HRR1) was defined as the absolute change from peak HR to HR 1 min post peak HR (HRR1 = peak HR – HR at 1 min post peak HR) (15, 16, 31). Similarly, HRs of recovery at 2 min (HRR2), 3 min (HRR3), 4 min (HRR4), and 5 min (HRR5) were calculated as the absolute change from peak HR to HRs 2, 3, 4, and 5 min, respectively, post-peak HR (31).

### Variables Measurements

The variables measurements were obtained at each visit (baseline, year 1, and year 2).

#### Anthropometric Measures

Body mass index (BMI) was calculated by dividing body weight in kilograms by the square of body height in meters.

#### Muscular Strength Performance Tests

Lower limb function was assessed with the timed stands test (TST), measuring the time in seconds required for 10 full stands from a sitting position in an armless chair.

Upper limb function was assessed with the Grippit® (AB Detektor, Göteborg Sweden), measuring the maximum and average (three measures) handgrip strength with an electronic dynamometer in Newton (N).

#### Cardiovascular Assessment

Resting blood pressure was measured after 5–10 min of rest in a seated position before the bicycle ergometer test with a sphygmomanometer and a stethoscope.

Pulse wave velocity (PWV) and aortic augmentation index (AIx) were measured at a constant room temperature and calculated using an oscillometric arteriograph (TensioMed, Budapest, Hungary). It has been described in detail in previous reports from PARA 2010 (6).

Transthoracic echocardiography examinations were performed using a commercially available ultrasound system (Vivid E9, GE Healthcare, Milwaukee, WI, USA). Standard two-dimensional and Doppler echocardiography was performed in keeping with EACVI/ASE recommendations (32). Digital loops were stored and analyzed offline by an experienced reader. Speckle-tracking echocardiography was used to measure LV global longitudinal strain using the apical four-, three-, and two-chamber views. Left atrial (LA) reservoir strain was used to represent atrial deformation in keeping with expert consensus (33).

#### Biomarkers

Plasma high-sensitivity C-reactive protein (hs-CRP) concentrations were measured by a particle-enhanced immunoturbidimetric method (Roche, France) with a measuring interval of 0.1–20 mg/ml. ESR was measured at baseline using the Westergren method in mm/h.

#### Questionnaires

A short version of the International Physical Activity Questionnaire (IPAQ) is a self-administered questionnaire collecting information about (i) MVPA in weekly metabolic equivalent of task-h (MET-h/week) and (ii) sedentary time (ST in h/d), undertaken over the past 7 days before the assessment (34). It was used to measure the physical activity of participants at baseline and at 1 and 2 years.

Quality of life was assessed with the EuroQol (EQ-5D 3L) visual analog scale (VAS), which measured health state the actual day with a line drawn from a box to the appropriate point on a vertical VAS from “worst imaginable health state” (= 0) to “best imaginable health state” (= 100) (23).

### Statistical Analysis

Data were expressed as mean (± SD) and frequencies (%). To determine statistically significant changes during the 2-year study protocol, a repeated-measured ANOVA on ranks was used with follow-up *post-hoc* comparisons. Wilcoxon's paired *t*-test was conducted for MVPA and ST data at years 1 and 2. Spearman rank correlation analyses were used to explore the relationship between HRR1/HRR2 and the different variables measured at baseline (Spearman correlogram). Machine learning models fit by elastic net linear regression provided regularized (shrunken) variable estimates and eliminated some variables from the set of predictors. These models were applied to predict HRR evolutions post-maximal exercise ECG using baseline, year 1, and 2 predictors chosen from significant associations in univariate analyses (Spearman correlograms). Eight-fold repeated crossvalidation was also applied to assess the performance of the model and to build a generalizable model. The predictive factors (magnitude ß-coefficient ≠ 0) were selected from each model. Correlations were determined between HRR1 (Models A and C)/HRR2 (Models B, D, and E) and (1) general characteristics of the participants: sex, age, anthropometric measures (BMI); (2) anaerobic and aerobic performances: TST (s), handgrip average and max (N), maximal aerobic power (W) (with % of expected); (3) cardiac parameters from resting and exercise ECG: resting HR (bpm), RMSSD (ms), % of expected maximal HR, HRs from exercise, recovery delta values (bpm), and cardiac parameters from echocardiography: LV strain (%) and LA reservoir strain (%); (4) vascular parameters from physical examination and exercise ECG: resting and mean blood pressures (mmHg), SBPs from exercise and recovery delta values (mmHg) and vascular parameters from arteriograph: PWV (m.s^−1^) and AIx (%); (5) inflammation biomarkers with CRP (mg.L^−1^); and (6) quality of life with EQ5D (/100).

Statistical analyses were performed using R (R Development Core Team, 2020) and SAS JMP Pro (JMP®, Version 16.1.0. SAS Institute Inc., Cary, NC, 1989–2021), where *p* < 0.05 was considered statistically significant. All tests were two-sided.

## Results

### Participants Characteristics

At baseline, the mean age was 60 ± 9.8 (41–73) years and 22 were women (88%). The mean DAS28 was 3.1 ± 1.2. Eighteen (72%) participants were taking biological RA medications, sixteen (64%) other DMARDs, eight (32%) were using non-steroidal anti-inflammatory drugs, and five (20%) cortisone. The mean values of ESR and LVEF were within normal ranges: 17 ± 11 mm and 59 ± 4% respectively. Out of the 25 participants, 22 (88%, 19 women) took part in the supported physical activity program (mean MVPA = 16.6 ± 5.4 MET-h/week and mean ST = 5.7 ± 2.3 h/d) in the first year; and 20 (80%, 18 women) continued the program autonomously (mean MVPA = 11.63 ± 4.6 MET-h/week and mean ST = 5.5 ± 2.6 h/d) during the second year. Exercise ECGs were all negative. Table 1 displays the general characteristics of the study population at baseline and at each yearly follow-up.

**Table 1:** General characteristics, static, and dynamic measures of the study cohort at baseline and yearly follow-ups.

**General characteristics**	**Baseline, mean** **(±SD)**	**Y1, mean** **(±SD)**	**Y2, mean** **(±SD)**
	***n* = 25**	***n* = 22**	***n* = 20**
Age (years)	60 (9.8)	60 (10)	59 (10)
BMI (kg/m^2^)	25.70 (4.7)	25.1 (4.5)	25.2 (4.2)
Hs-CRP (mg/l)	2.8 (2.5)	1.6 (1.8)	1.8 (1.4)
EQ5D (0–100)	73 (17)	75 (19)	77 (16)
**Static measures**			
HR rest (bpm)	70 (9)	67 (8)	67 (7.4)
RMSSD (ms)	**31.4 (13.4)**	**43.9 (16.9)**	**36.9 (15.3)**
Resting SBP (mmHg)	127 (10)	126 (10)	124 (12)
Resting mean blood pressure (mmHg)	**99.9 (13.4)**	**86 (11.5)**	**87.5 (11.2)**
LV global longitudinal strain (%)	20.35 (3)	19.56 (3.1)	20.7 (2.8)
LA reservoir strain (%)	31.5 (8.1)	33.12 (9.7)	34.6 (9.9)
PWV (m/s)	10.8 (2.2)	9.8 (2.4)	11.4 2.7)
Aortic augmentation index	36.1 (10.9)	35.8 (10.2)	38 (10.8)
**Dynamic measures**			
Handgrip average (N)	196 (119)	200 (115)	217 (114)
Handgrip max (N)	239 (136)	235 (131)	257 (132)
TST (s)	**19 (6)**	**16 (6)**	**14 (3)**
MAP (W)	168 (48)	181 (46)	176 (45)
Theoretical % max HR	100 (7)	102 (7)	99 (7)
Theoretical % MAP	134 (23)	145 (24)	144 (12)
HR exercise delta (2 min-rest) (bpm)	46 (14)	38 (11)	45 (11)
HR increase (max-rest) (bpm)	84.4 (13.7)	89 (13)	86 (12)
SBP exercise delta (2 min-rest) (mmHg)	26.4 (14)	26.5 (16.6)	28.8 (15.5)
SBP exercise delta (max-rest) (mmHg)	56.3 (11.1)	56.7 (12.7)	52.3 (18)
SBP recovery delta (max-2 min) (mmHg)	**28.9 (11.8)**	**32.4 (15.1)**	**21.5 (9.2)**
SBP recovery delta (max-5 min) (mmHg)	**47.6 (14)**	**49.7 (18)**	**42 (13.3)**

*ANOVA on ranks analysis was conducted to determine statistically significant changes during the 2-year study protocol. Bold font style indicates a statistically significant relationship between column and row parameters (p < 0.05). SD, standard deviation; BMI, body max index; Hs-CRP, Plasma high-sensitivity C-reactive protein; EQ5D, EuroQol 5-dimensions; HR, Heart rate; RMSSD, Root mean square of the successive differences; SBP, Systolic blood pressure; LV, Left ventricular; LA, Left atrial; PWV, Pulse wave velocity; TST, Timed stands test; MAP, Maximal aerobic power*.

**Figure 1:**
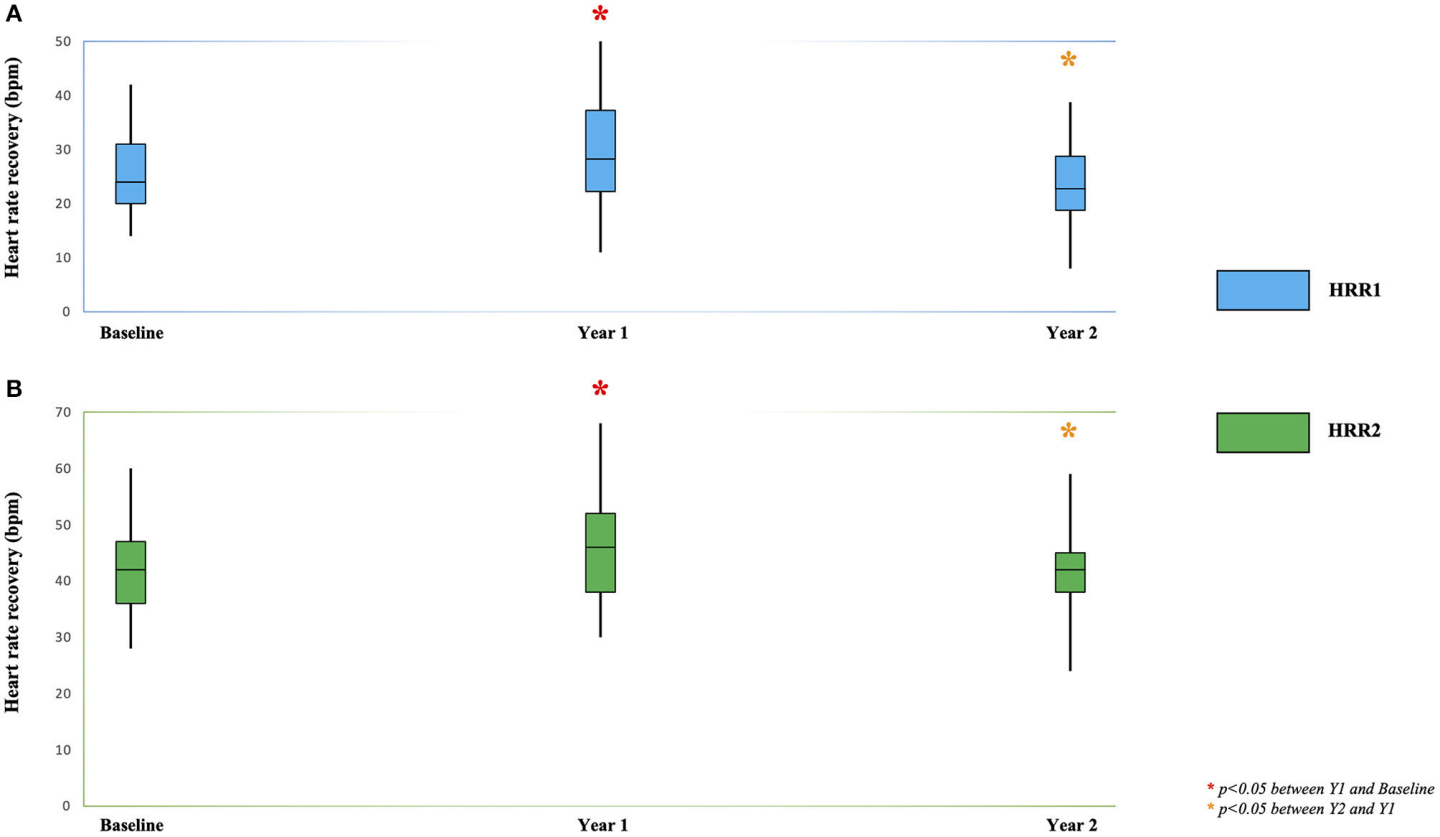
Heart rate recovery at 1 min: HRR1 **(A)** and 2 min: HRR2 **(B)** during the 2-year physical activity program in participants with RA. ANOVA on ranks analysis was conducted to determine statistically significant changes during the 2-year study protocol. *p < 0.05 between Y1 and Baseline. *p < 0.05 between Y2 and Y1.

A statistically significant increase was observed in the first year (compared to baseline), then a statistically significant decrease was observed in the second year (compared to year 1) for HRR1 (*p* = 0.02 and *p* = 0.01), HRR2 (*p* = 0.03) (Table 2; Figure 1; Supplementary Figure 1), RMSSD (*p* = 0.03 and *p* = 0.04), SBP recovery index (*p* = 0.01 for 2- and 5-min recovery from exercise) (Table 1; Supplementary Figure 2) and MVPA (*p* = 0.04 between years 1 and 2).

**Table 2:** Heart rate recovery (HRR) measures of the study cohort at baseline and yearly follow-ups.

**Heart rate**	**Baseline, mean (±SD)**	**Y1, mean (±SD)**	**Y2, mean (±SD)**
**recovery**,	***n* = 25**	***n* = 22**	***n* = 20**
**HRR (bpm)**			
HRR1	**25 (8)**	**28 (10)**	**24 (9)**
HRR2	**42 (9)**	**47 (14)**	**43 (11)**
HRR3	55 (11)	57 (13)	55 (12)
HRR4	60 (11)	63 (11)	61 (12)
HRR5	62 (12)	66 (12)	64 (11)

*ANOVA on ranks analysis was conducted to determine statistically significant changes during the 2-year study protocol. Bold font style indicates a statistically significant relationship between column and row parameters (p > 0.05)*.

A significant improvement in resting mean blood pressure (*p* = 0.03) and TST (*p* = 0.04) was observed over the entire study period. All these associations remained significant even after adjusting for the non-sphericity of the data (Table 1).

### Predictions

In order to predict (1) the significant increase of HRR1 and HRR2 at 1 year (from baseline) and (2) the significant decrease of HRR1 and HRR2 at 2 years (from year 1) of the PARA program (Table 2; Figure 1; Supplementary Figure 1), the most clinically relevant variables were subsequently entered in elastic net linear regression models (Table 3; Supplementary Figure 3).

**Table 3:** The results of five elastic net linear regression models applied to predict heart rate recovery (HRR) 1 and 2 increases (Models A and B) and decreases (Models C, D, and E) post-maximal exercise ECG using the baseline, year 1, and year 2 predictors as independent variables.

**Parameters**	**Model A**	**Model B**	**Model C**	**Model D**	**Model E**
**General characteristics**	**Beta coefficient (** ***standard error*** **)**				
Age (years)	-	-	-	-	-
BMI (kg/m^2^)	−0.88 (*3.46*)	-	-	-	-
Hs-CRP (mg/l)	-	3.38 (*9.32*)	-	-	-
EQ5D (0–100)	-	-	-	-	-
**Static measures**	-	-	-	-	-
HR rest (bpm)	-	-	-	-	-
RMSSD (ms)	-	-	-	-	-
Resting SBP (mmHg)	-	-	-	-	-
Resting mean blood pressure (mmHg)	-	-	-	-	-
LV global longitudinal strain (%)	-	-	-	-	1.39 (*113.15*)
LA reservoir strain (%)	-	-	-	-	-
PWV (m/s)	-	-	-	-	-
Aortic augmentation index	-	-	-	-	-
**Dynamic measures**	-	-	-	-	-
Handgrip average (N)	-	-	1.34 (*4.77*)	-	-
Handgrip max (N)	-	-	-	-	-
TST (s)	0.47 (*5.99*)	-	-	-	−3.10 (*173.45*)
MAP (W)	-	-	-	-	-
Theoretical % max HR	-	1.26 (*10.48*)	-	-	-
Theoretical % MAP	-	-	-	-	-
HR exercise delta (2 min-rest) (bpm)	-	-	-	-	-
HR increase (max-rest) (bpm)	-	-	-	-	-
SBP exercise delta (2 min-rest) (mmHg)	1.54 (*5.81*)	-	−1.71 (*4.93*)	−0.97 (*2.99*)	-
SBP exercise delta (max-rest) (mmHg)	0.07 (*4.92*)	-	-	-	-
SBP recovery delta (max-2 min) (mmHg)	1.88 (*4.11*)	3.99 (*5.68*)	-	-	-
SBP recovery delta (max-5 min) (mmHg)	0.19 (*6.91*)	-	-	-	-

*The predictors with a non-zero beta coefficient are represented in each model. The dashes correspond to zero beta predictors. The predictive factors set was chosen based on the eight-fold cross-validation approach*.

*BMI, body max index; Hs-CRP, Plasma high-sensitivity C-reactive protein; EQ5D, EuroQol 5-dimensions; HR, Heart rate; RMSSD, Root mean square of the successive differences; SBP, Systolic blood pressure; LV, Left ventricular; LA, Left atrial; PWV, Pulse wave velocity; TST, Timed stands test; MAP, Maximal aerobic power*.

**Figure 2:**
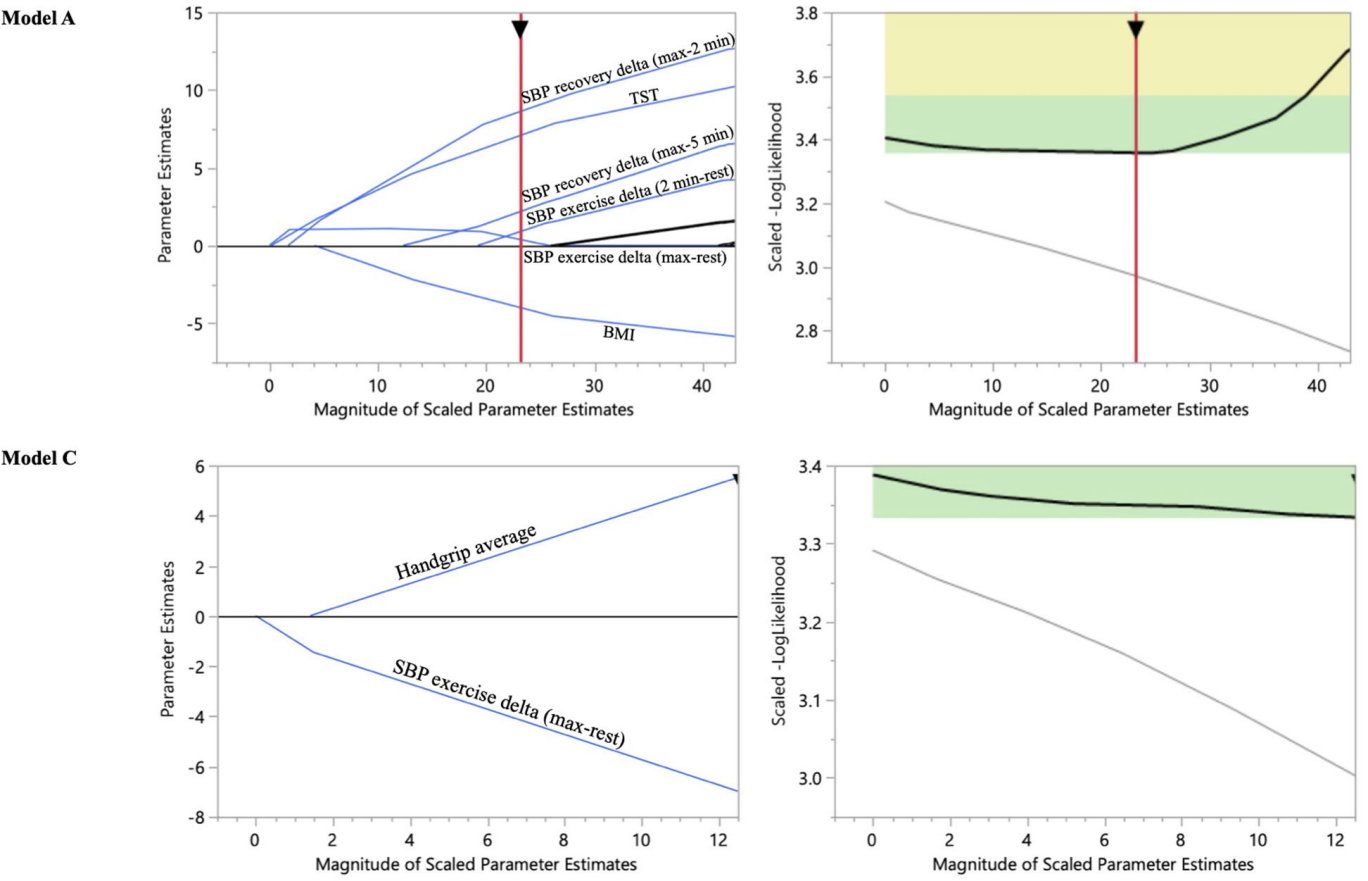
Solution path trace plot applied to predict HRR1 increase and decrease post-maximal exercise ECG using the baseline and year 1 predictors as independent variables: Models A and C. Each line corresponds to a predictor variable. As regularization is applied to the model (from right to left in the trace plot), less important coefficients get smaller, and some are completely removed from the model (the ones that have a beta coefficient of 0 and where the line for the predictor intersects with the x-axis before the optimal solution is reached). A generalizable model was built with an eight-fold cross validation to determine the model which provides a global minimum (within the green rectangle) for the scaled-log likelihood value among the validation folds.

Five models were built for predicting five types of outcomes: (1)- the increase of HRR1 (Model A) and (2)- HRR2 (Model B) during the first year of the program, from predictors obtained at baseline, (3)- the decrease of HRR1 (Model C), and (4)- HRR2 (Model D) during the second year of the program, from predictors obtained at year 1 and year 2 for HRR2 (Model E). These five elastic net regression models were retained in a total of 10 of 24 candidate predictors for HRR changes (Table 3; Figure 2; Supplementary Figure 4).

The SBP response to exercise at baseline was the best predictor for an increase of HRR1 (Model A) and HRR2 (Model B). Indeed, SBP recovery at 2 min was positively correlated with HRR1 (Model A: ß-coefficient = 1.88) and HRR2 (Model B: ß-coefficient = 3.99) increase (Table 3; Figure 2; Supplementary Figure 4).

For the changes at year 1, SBP increase at baseline exercise, lower BMI, and higher TST were the other predictive covariates of HRR1 increase (Model A: ß-coefficient = 1.54, −0.88, and 0.47 respectively) (Table 3; Figure 2). Hs-CRP and % theoretical max were the other predictive covariates of HRR2 increase (Model B: ß-coefficient = 3.38 and 1.26, respectively) (Table 3; Supplementary Figure 4).

For the changes at year 2, SBP response to exercise at year 1 was the best predictor of HRR1 (Model C) and HRR2 (Model D) decrease (Table 3; Figure 2; Supplementary Figure 4). Indeed, the SBP exercise delta (at year 1) was negatively correlated with HRR1 (Model C: ß-coefficient = −1.71) and HRR2 (Model D: ß-coefficient = −0.96) decrease (at year 2) (Table 3; Figure 2; Supplementary Figure 4). Handgrip was the only other covariate of HRR1 decrease (Model C: ß-coefficient = 1.34) (Table 3; Figure 2). TST and LV strain were the predictors at year 2 of HRR2 decrease during the second part of the program (Model E: ß-coefficient = −3.1 and 1.39, respectively) (Table 3; Supplementary Figure 4).

## Discussion

The results of this study identified a parallel relation between the changes in levels of physical activity with ANS activity in RA. In particular, a significant improvement after 1 year of supervised physical activity and a decrease toward baseline during the following year with less intense physical activity is noted, as illustrated in Supplementary Figure 5. Integrating all plausible factors affecting ANS activity, the present machine learning elastic net regression models identified BMI, CRP, and TST as well as HR and SBP responses to exercise as baseline predictors of the exercise associated ANS improvement in RA (Supplementary Figure 5). Taken together, these findings extend the well-established positive effects of exercise in RA to measures and predictors of ANS, with potential clinical implications for CVD risk.

The first 2 min of HRR is validated and commonly used ANS measures (31). A slow decrease of HRR after exercise indicates autonomic dysfunction or at least a non-optimal ANS and is an independent predictor of CVD and all-cause mortality (18). Also, an impaired HRR from the first minute after exercise ECG is a predictor of overall mortality (19, 35). In this study, the HRRs 1–5 were largely above the normal cut-off of 12 beats per minute (17), but lower than the HRR observed in larger population-based cohorts (36). The absolute HRR 1 and 2 in this work were similar to previous work in patients with RA after maximal treadmill testing (20) supporting an autonomic dysfunction in RA. Cardiac rehabilitation increases HRR, which is associated with improved outcomes (37). The reversibility of the autonomic dysfunction in RA has *hitherto* remained unexplored. Therefore, we next assessed HRR after a 2-year physical activity program in RA participants (20) to establish the possibility to improve HRR and to determine the main predictive factors associated with HRR in RA.

Importantly, an improved ANS function in RA after the first year of supervised physical activity was demonstrated in this study by significant increases of HRR at 1 and 2 min after maximal exercise. Beneficial cardiovascular effects of exercise interventions in RA are well-established (6, 8–11, 20), and our results extend these findings to a potential link *via* the enhancement of parasympathetic modulation, which was further supported by the significant increase in RMSSD in the present study.

Elastic regression modeling with eight-fold cross validation allowed us to identify blood pressure response to exercise, lower BMI, and higher muscular strength at baseline as predictors of HRR increase during the first year of the PARA 2010 physical activity program. These findings point to the possibility to identify responders and non-responders to physical activity intervention programs in RA.

Both the SBP increase at exercise and the decrease at recovery predicted the HRR increase in response to the PARA2010 intervention in RA. Previous studies have established a relationship between HRR and SBP, but provided contradictory conclusions if SBP changes during exercise are a consequence or cause of the observed HRR (38–42). In this study, SBP recovery followed the pattern of HRR and increased in the first year. The rise and recovery of SBP with exercise is predominantly caused by an increase in cardiac output, being dependent on HR and systolic function (28, 42), which was normal in this study. In RA, increased arterial stiffness (7, 43), diastolic dysfunction (9), and a non-optimal ventricular-arterial coupling (6) may affect the SBP increase and recovery after exercise (28, 44). In addition to SBP responses, positive predictive factors for improved HRR by exercise intervention identified by elastic net regression analysis included also strength and a low BMI at baseline. These observations indicate conditions giving the optimal conditions for improving ANS function by exercise. In contrast, higher CRP predicted an increase in HRR. Exposure to inflammation affects the cardiovascular system, as demonstrated by the disease duration as a major determinant of vascular function in RA (7). There is, in addition, a close relation and possible interregulation between inflammation and ANS activity. The observation that participants with higher CRP exhibited a larger improvement in HRR supports that inflammatory conditions indicate a larger beneficial potential of physical activity.

The observed ANS improvement at year 1 was not preserved over time since HRR was significantly decreased at year 2 compared with year 1 in this study, despite a level of physical activity that continued to overreach the WHO recommendations (>2x in the first year and 1.5x in the second year) (24). These results suggested a high efficient threshold of exercise training to obtain an adapted autonomic response, potentially optimizing the cardiovascular response and improving cardiovascular outcomes. The slightest decrease in physical activity (even over physical activity recommendations) leads to a sympathovagal balance shifting toward less parasympathetic activity. Finally, assessment of the last available cardiovascular characteristics identified a negative association of HRR increase with TST decrease and a positive association with normal-to-high values of LV strain at year 2. Thus, a substantial gain of muscular strength (lower limb with TST and upper limb with handgrip) and a normal LV systolic deformation (absence of subclinical cardiac dysfunction) predicted a better response to exercise after the 2-year physical activity program. Both are strong cardiovascular prognostic factors (9, 45–47), associated with a reduction in resting blood pressure (48, 49). In addition to being significantly correlated at baseline, the significant correlations between autonomic and vascular functions at 1 and 2 years indicate that these two variables are closely related to each other and maybe part of the indirect effects of physical activity increase during the first year and physical activity decrease in the second year.

The bidirectional changes in HRR over increasing and decreasing physical activity may be used to follow a physical activity intervention in RA to identify non-responder and non-adherent participants with potential hypertension risk, despite normal resting blood pressure and normal exercise ECG findings. From a practical standpoint, exercising ECG and extending it for only several minutes into exercise recovery provides a unique, minimally invasive means to assess HR and blood pressure responses to exercise to indirectly determine participant's response or adherence to physical activity and to predict the CVD risk (28, 50), and also mainly to detect hypertension early (39, 42, 51). It was already reported in the literature that a delayed SBP decrease post-exercise was more accurate than ST segment depression for the diagnosis of coronary artery disease (CAD) (39, 52, 53). Early identification of high cardiovascular risk could improve the effectiveness of prevention in RA by targeting patients who will benefit the most from lifestyle interventions (exercise, diet, and antihypertensive drugs). The present results suggest that ANS upregulation by a high (≥7.5 MET-h/week) and regular (≥5 days/7) MVPA training level may be key for successful aging. Obviously, the support group sessions according to social cognitive theory improved adherence of RA participants in the first year (supervised physical activity). The outcomes of this study could provide new insight into the relationship between SBP response to exercise and ANS modulation.

Major strengths of this study include the prospective longitudinal design with close follow-up of the participant's adherence to the physical activity program and with the repeated physiological parameter assessments (23). The longitudinal follow-up allowed to assess causality between physiological parameters according to the steps of the PARA cohort study, which are 0–1 year: whole program of physical activity; and 1–2 years: autonomously physical activity (23). No subject of the study had medication that could affect autonomic function (anticholinergics, cholinesterase antagonists, adrenoreceptor agonists, and adrenoreceptor antagonists). Most of the participants were able to perform the exercise ECG to their maximum cardiorespiratory ability, i.e., until exhaustion (the mean theoretical % of MAP was exceeded by 34–45% each time), despite problems with their joints, which could have influenced their HRR.

Certain limitations should also be acknowledged. First, the relatively small sample size is mainly made up of Swedish women (88%). It is the same population described in the two previous studies from the team, but four participants were excluded due to incomplete data concerning exercise ECG (6, 9). However, the use of a machine-learning algorithm with the elastic net linear regression models permits the identification of predictive factors among an important number of parameters (*p*) in relation to a small sample (*n*) of participants (*p* > *n*). Nested repeated crossvalidation was used for the models in the study, which provides an unbiased estimation of the performance of the prediction model (54). Compared with a classical linear regression method, the elastic net linear regression model increases the robustness, simplicity, and accuracy of such results in this context (55, 56). However, standard errors are large with the small sample. Second, the study did not have a comparison group, but the aim of the work was to examine the effects of a physical activity intervention on HRR in this population. Third, difficulties, even with experience, to define the values of diastolic blood pressure when it comes to defining or recognizing the fifth Korotkoff sound during exercise ECG (57). Also, a common measure of SBP recovery is defined in the literature as the ratio of the SBP obtained in the third minute of the recovery period to either the peak-exercise SBP or the SBP in the first minute of the recovery period after exercise ECG (39, 42, 58). Unfortunately, SBP was measured at 2 and 5 min recovery from exercise in the PARA study, which is generally performed in clinical research in practice (51, 57). Finally, as participants were tasked to report their level of physical activity, the reporting bias should be acknowledged even if it was the most validated physical activity questionnaire (59). Future studies should be to use objective methods such as accelerometry to assess physical activity, mainly when this activity is practiced in autonomy. A more precise evaluation of physical activity and biological data could determine if exercise leads to a sympathovagal balance shifting toward a more parasympathetic activity or less sympathetic activity (60).

## Conclusion

Autonomic nervous system activity *via* HRR measurement can be a relevant marker of the effectiveness of physical activity recommended in patients with RA at high risk of CVD. Exercise ECG can serve as a simple minimally invasive means to assess blood pressure responses to exercise and recovery, which are the main predictive factors of HRR evolutions during a physical activity program. HRR and SBP recovery, associated with muscular strength performance tests, can mainly be used to assess sympathovagal balance and to quantify the degree of autonomic dysfunction in order to detect CVD risk early. These results can guide future recommendations for RA patients and may improve adherence to regular physical activity programs and thus their cardiovascular health.

## Data Availability Statement

The original contributions presented in the study are included in the article/Supplementary Material, further inquiries can be directed to the corresponding author.

## Ethics Statement

The studies involving human participants were reviewed and approved by the Stockholm Regional Ethics Committee (Reference Number 2012/769-32). The patients/participants provided their written informed consent to participate in this study.

## Author Contributions

DH, PS, CO, IL, and MB: conception and design of the work, interpretation, and draft of the manuscript and substantial revision. DH, AV, CF, BN, and MB: acquisition. DH, PS, and MB: analysis. All authors have approved the manuscript.

## Funding

PARA 2010 was supported by grants from AFA Insurance (Grant Number 100172), The Swedish Research Council, Combine Sweden, The Swedish Rheumatism Foundation, and The KI National Postgraduate School of Health Care Sciences. DH was supported by the Foundation for Geriatric Diseases at Karolinska Institutet (Grant Number FS-2021:0006), the Auvergne-Rhone-Alpes Region (Grant Number 20 006296 01), the GHT-Loire public hospital funds, and the European Society of Cardiology Council on Basic Cardiovascular Science (Grant Number 000085188), French Institute in Sweden (TOR scholarship SCIEN 23/21). PS was supported by the Clinical Scientist Training Programme (CSTP) at Karolinska Institutet. MB was supported by the Swedish Research Council (Grant Number 2019-01486), the Swedish Heart and Lung Foundation (Grant Number 20180571), and King Gustaf V and Queen Victoria Freemason Foundation. The funding sources had no role in the design and conduct of the study, collection, management, analysis, and interpretation of the data, preparation, review, or approval of the manuscript, and decision to submit the manuscript for publication.

## Conflict of Interest

The authors declare that the research was conducted in the absence of any commercial or financial relationships that could be construed as a potential conflict of interest.

## Publisher's Note

All claims expressed in this article are solely those of the authors and do not necessarily represent those of their affiliated organizations, or those of the publisher, the editors and the reviewers. Any product that may be evaluated in this article, or claim that may be made by its manufacturer, is not guaranteed or endorsed by the publisher.
