# Supplementary material for: Cardiovascular Autonomic Function Changes and Predictors During a 2-Year Physical Activity Program in Rheumatoid Arthritis: A PARA 2010 Substudy
Source: Front Med (Lausanne). 2021 Dec 15;8:788243. doi: 10.3389/fmed.2021.788243 (PMC8717774; doi:10.3389/fmed.2021.788243)
Supplement: Supplementary file 1 [file Data_Sheet_1.pdf]

Supplementary Material

Supplementary Figure 1. Heart rate recovery at 1 (HRR1), 2 (HRR2), 3 (HRR3), 4 (HRR4) and 5 (HRR5) minutes during 2-year physical activity program in participants with RA.
ANOVA on ranks analysis was conducted to determine statistically significant changes during the 2-year study protocol. * *p<0.05 between Y1 and Baseline.* * *p<0.05 between Y2 and Y1*

Supplementary Figure 2. Blood pressure response to exercise during 2-year physical activity program in participants with RA: systolic blood pressure (SBP) exercise deltas (2 min-rest and max-rest) and recovery deltas (max-2 min and max-5 min) at Year 1 and Year 2 as compared to Baseline.
ANOVA on ranks analysis was conducted to determine statistically significant changes during the 2-year study protocol. * *p<0.05 between Y1 and Baseline.* * *p<0.05 between Y2 and Y1*

Supplementary Figure 3. Correlogram of Spearman's rank correlation coefficients between HRR1/HRR2 and the different variables measured at baseline.
*Color indicates whether the correlation is positive (blue) or negative (red). Size and darkness of the circles indicate the strength of the correlations, with stronger correlations being larger and darker than weaker ones. *p<0.05*

Supplementary Figure 4. Solution path trace plot applied to predict HRR2 increase and decrease post maximal exercise ECG using the year 1 and year 2 predictors as independent variables: Models B, D and E. *Each line corresponds to a predictor variable. As regularization is applied to the model (from right to left in the trace plot), less important coefficients get smaller, and some are completely removed from the model (the ones that have a beta coefficient of 0 and where the line for the predictor intersects with the x-axis before the optimal solution is reached). A generalizable model was built with a 8-fold cross validation in order to determine the model which provides a global minimum (within the green rectangle) for the scaled -log likelihood value among the validation folds.*

Supplementary Figure 5. Graphic illustration of the main results from the PARA 2010 substudy
WHO: World Health Organization; BP: Blood pressure; TST: Timed stands test; HRR: Heart rate recovery; RMSSD: Root mean square of the successive differences; SBP: Systolic blood pressure; % th max HR: % of theoretical maximal heart rate; BMI: body max index; LV: Left ventricular; Hs-CRP: High-sensitivity C-reactive Protein

Supplementary Figure 1. Heart rate recovery at 1 (HRR1), 2 (HRR2), 3 (HRR3), 4 (HRR4) and 5 (HRR5) minutes during 2-year physical activity program in participants with RA.
ANOVA on ranks analysis was conducted to determine statistically significant changes during the 2-year study protocol. * $p<0.05$ *between Y1 and Baseline.* * $p<0.05$ *between Y2 and Y1*

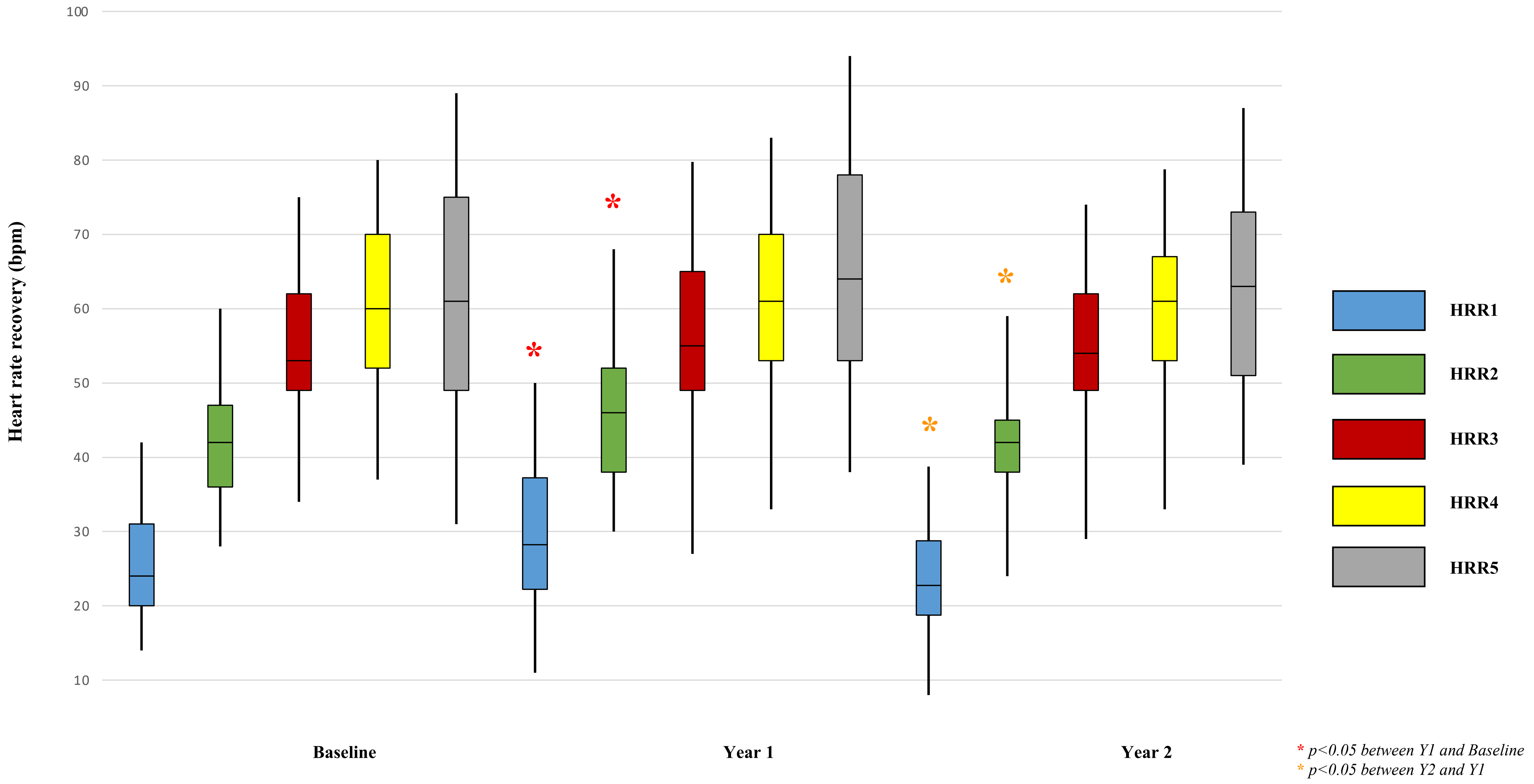
Heart rate recovery (bpm)
100
90
80
70
60
50
40
30
20
10
Baseline
Year 1
Year 2
HRR1
HRR2
HRR3
HRR4
HRR5
* p<0.05 between Y1 and Baseline
* p<0.05 between Y2 and Y1

Supplementary Figure 2. Blood pressure response to exercise during 2-year physical activity program in participants with RA: systolic blood pressure (SBP) exercise deltas (2 min-rest and max-rest) and recovery deltas (max-2 min and max-5 min) at Year 1 and Year 2 as compared to Baseline. ANOVA on ranks analysis was conducted to determine statistically significant changes during the 2-year study protocol. ***** *$p<0.05$ between Y1 and Baseline.* ***** *$p<0.05$ between Y2 and Y1*

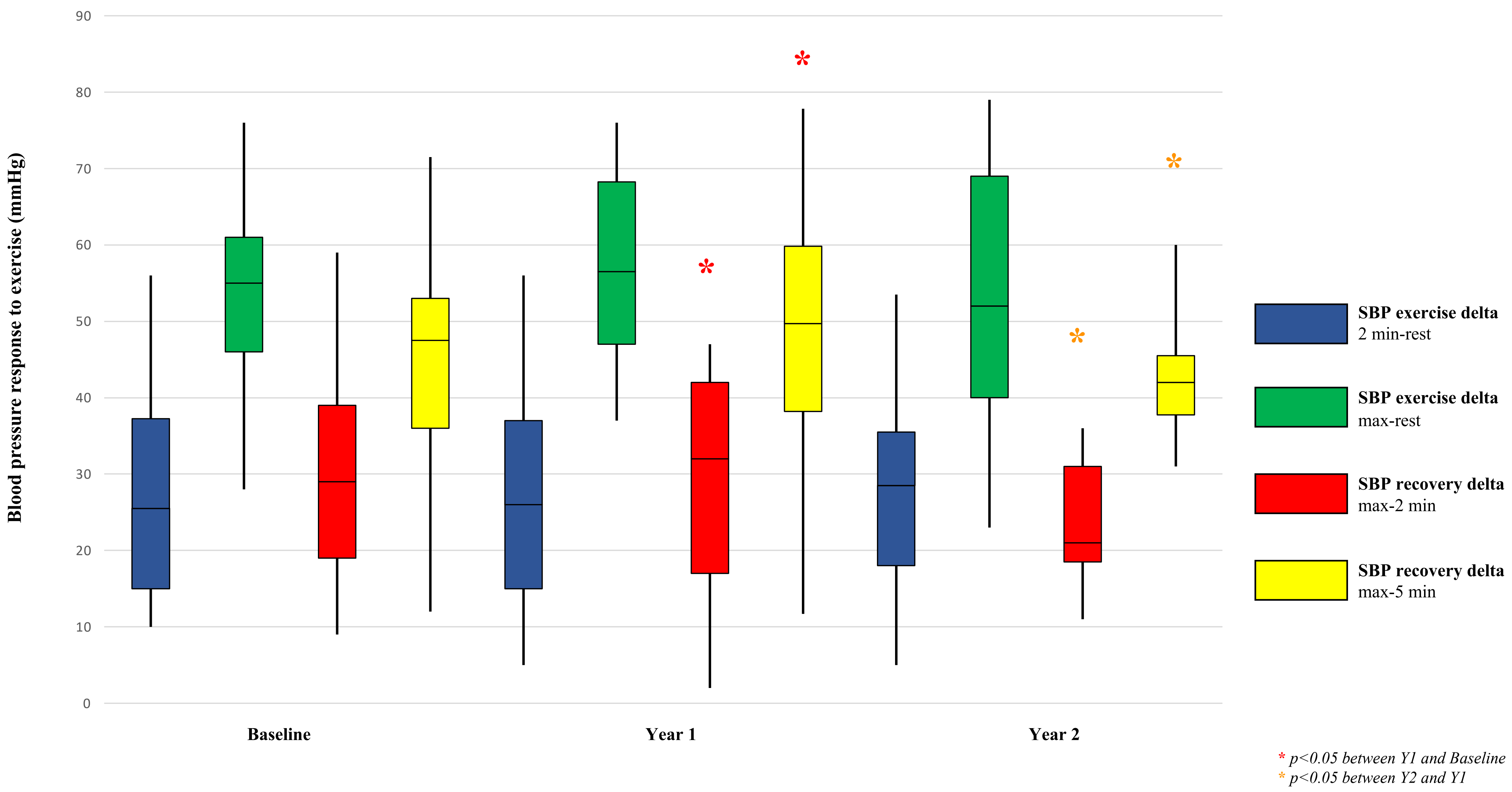
Blood pressure response to exercise (mmHg)
0
10
20
30
40
50
60
70
80
90
Baseline
Year 1
Year 2
SBP exercise delta
2 min-rest
SBP exercise delta
max-rest
SBP recovery delta
max-2 min
SBP recovery delta
max-5 min
* p<0.05 between Y1 and Baseline
* p<0.05 between Y2 and Y1

Supplementary Figure 3. Correlogram of Spearman's rank correlation coefficients between HRR1/HRR2 and the different variables measured at baseline.
Color indicates whether the correlation is positive (blue) or negative (red). Size and darkness of the circles indicate the strength of the correlations, with stronger correlations being larger and darker than weaker ones. *$p<0.05$

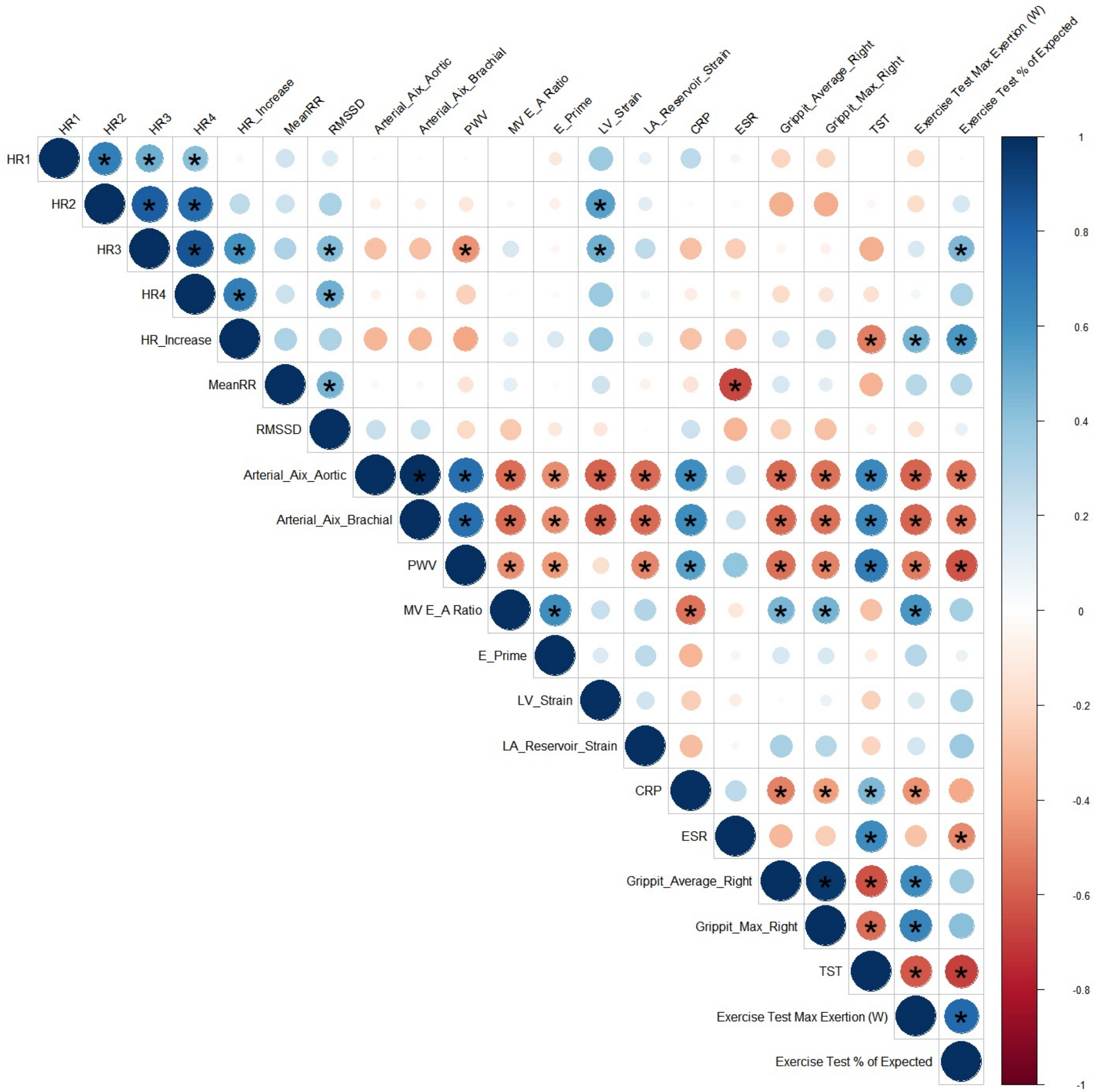
HR1
HR2
HR3
HR4
HR_Increase
MeanRR
RMSSD
Arterial_Aix_Aortic
Arterial_Aix_Brachial
PWV
MV E_A Ratio
E_Prime
LV_Strain
LA_Reservoir_Strain
CRP
ESR
Grippit_Average_Right
Grippit_Max_Right
TST
Exercise Test Max Exertion (W)
Exercise Test % of Expected
1
0.8
0.6
0.4
0.2
0
-0.2
-0.4
-0.6
-0.8
-1

Supplementary Figure 4. Solution path trace plot applied to predict HRR2 increase and decrease post maximal exercise ECG using the year 1 and year 2 predictors as independent variables: Models B, D and E. *Each line corresponds to a predictor variable. As regularization is applied to the model (from right to left in the trace plot), less important coefficients get smaller, and some are completely removed from the model (the ones that have a beta coefficient of 0 and where the line for the predictor intersects with the x-axis before the optimal solution is reached). A generalizable model was built with a 8-fold cross validation in order to determine the model which provides a global minimum (within the green rectangle) for the scaled -log likelihood value among the validation folds.*

Model B

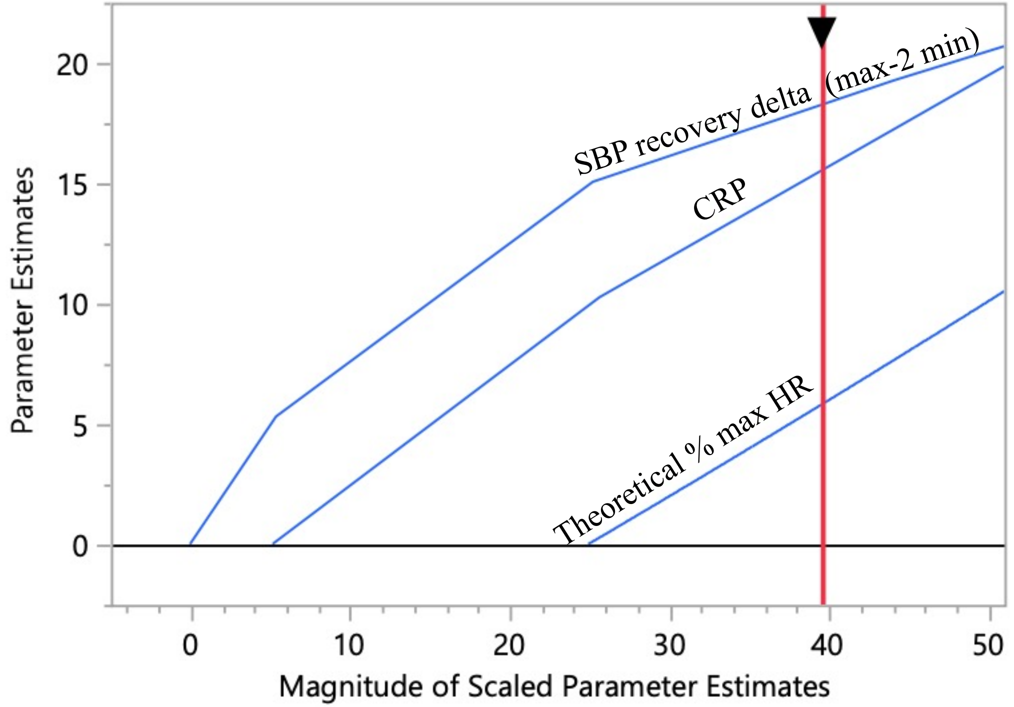

Parameter Estimates
SBP recovery delta (max-2 min)
CRP
Theoretical % max HR
Magnitude of Scaled Parameter Estimates

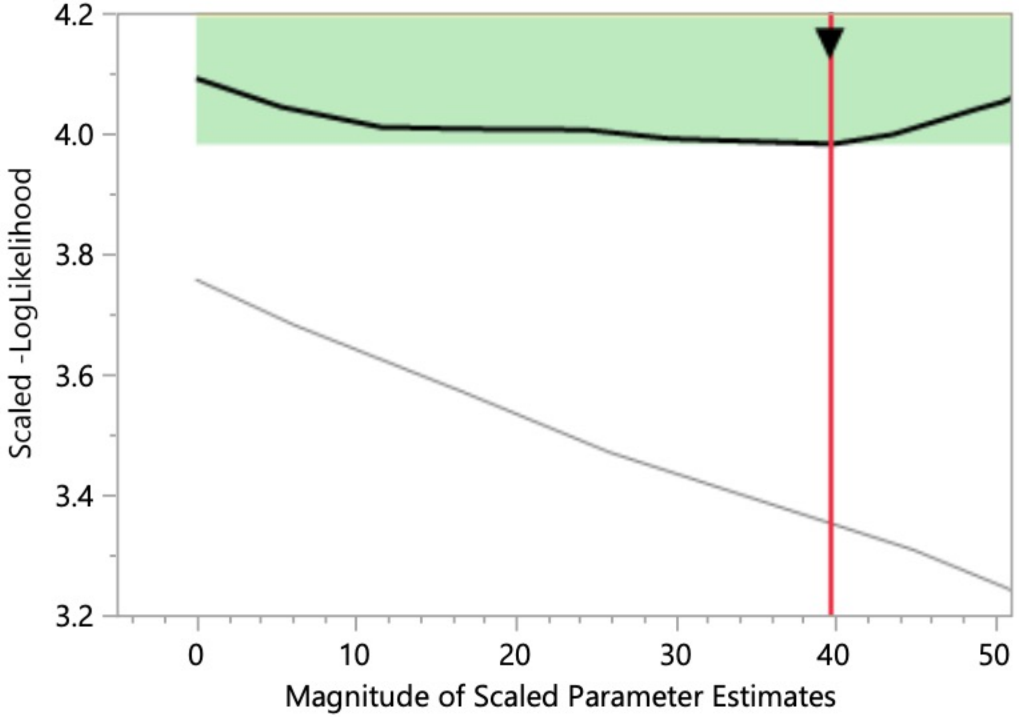

Scaled -LogLikelihood
Magnitude of Scaled Parameter Estimates

Model D

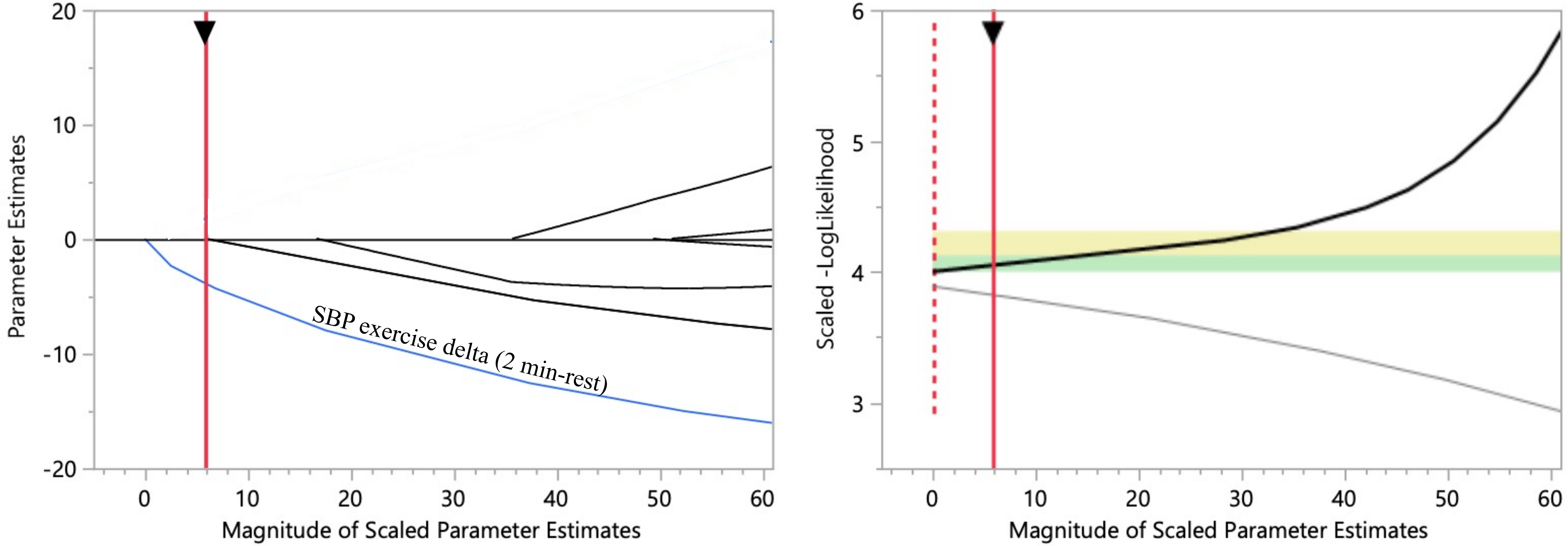
Parameter Estimates
20
10
0
-10
-20
SBP exercise delta (2 min-rest)
0
10
20
30
40
50
60
Magnitude of Scaled Parameter Estimates
Scaled -LogLikelihood
6
5
4
3
0
10
20
30
40
50
60
Magnitude of Scaled Parameter Estimates

Model E

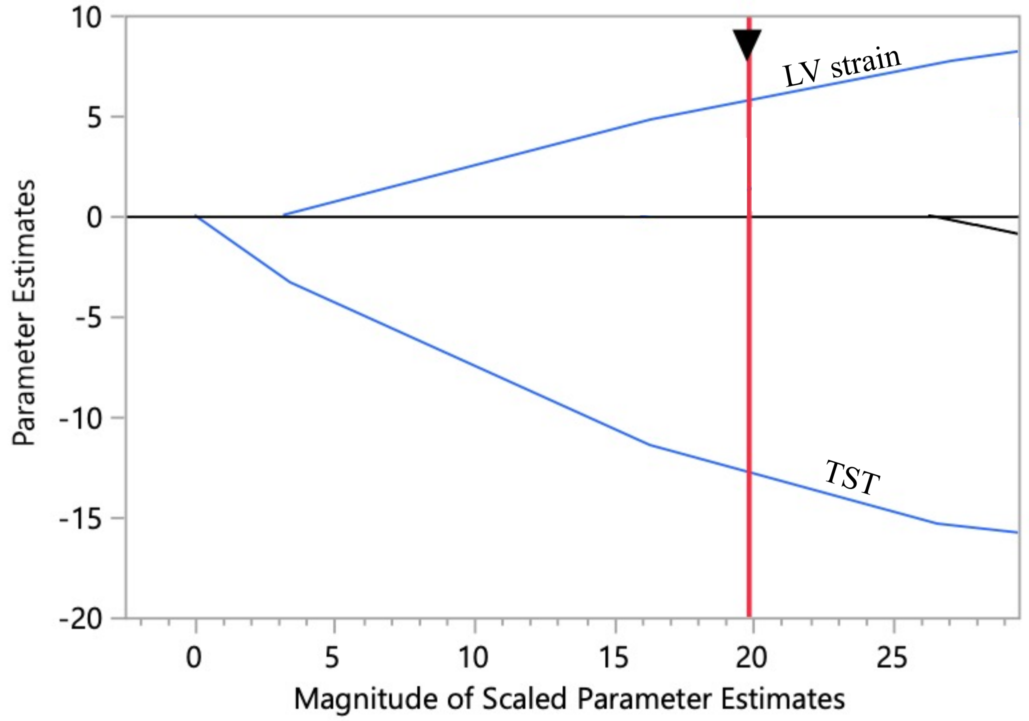

Parameter Estimates
10
5
0
-5
-10
-15
-20
LV strain
TST
0
5
10
15
20
25
Magnitude of Scaled Parameter Estimates

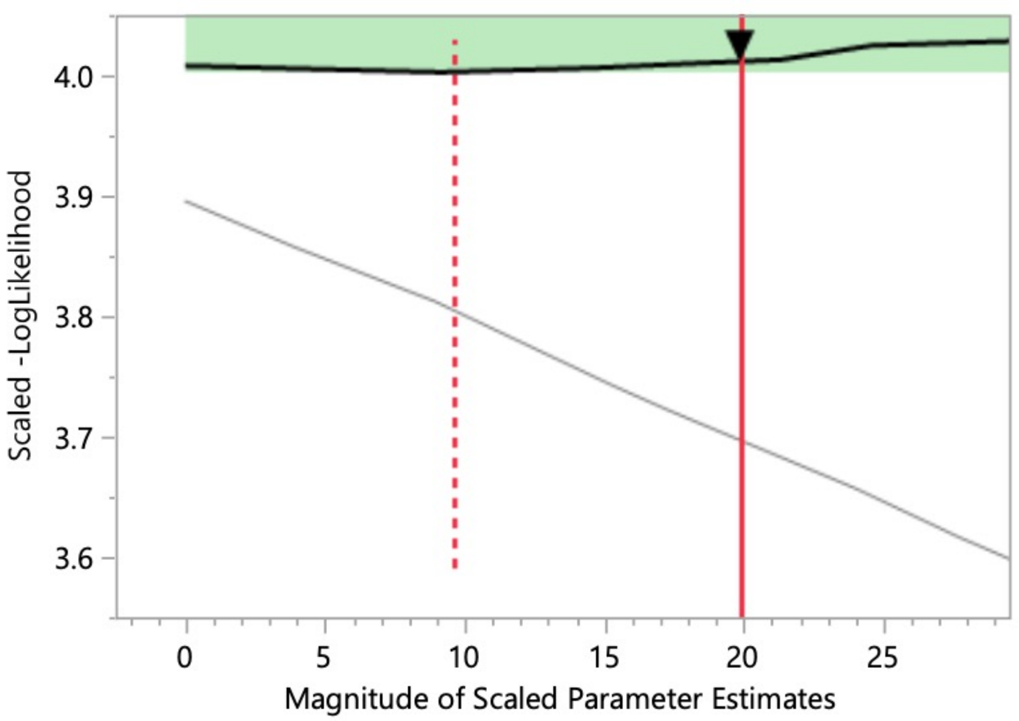

Scaled -LogLikelihood
4.0
3.9
3.8
3.7
3.6
0
5
10
15
20
25
Magnitude of Scaled Parameter Estimates

Supplementary Figure 5. Graphic illustration of the main results from the PARA 2010 substudy
WHO: World Health Organization; BP: Blood pressure; TST: Timed stands test; HRR: Heart rate recovery; RMSSD: Root mean square of the successive differences; SBP: Systolic blood pressure; % th max HR: % of theoretical maximal heart rate; BMI: body max index; LV: Left ventricular; Hs-CRP: High-sensitivity C-reactive Protein

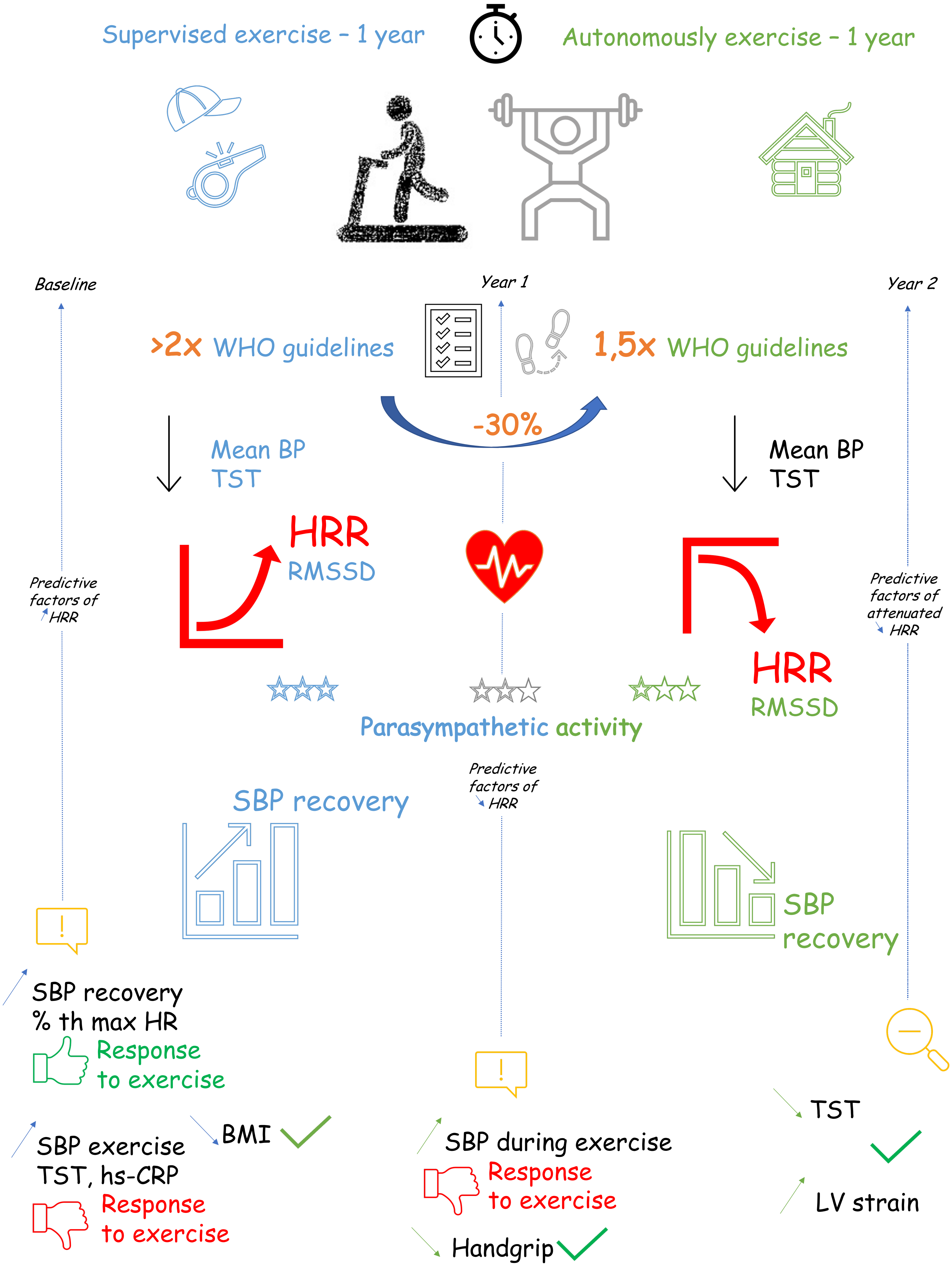
Supervised exercise – 1 year
Autonomously exercise – 1 year
Baseline
Year 1
Year 2
>2x WHO guidelines
1,5x WHO guidelines
-30%
Mean BP
TST
Mean BP
TST
HRR
RMSSD
HRR
RMSSD
Predictive factors of HRR
Predictive factors of attenuated HRR
Parasympathetic activity
Predictive factors of HRR
SBP recovery
SBP recovery
SBP recovery
% th max HR
Response to exercise
SBP exercise
TST, hs-CRP
BMI
Response to exercise
SBP during exercise
Response to exercise
Handgrip
TST
LV strain
